# The epidemiology of haematological cancers in Sarawak, Malaysia (1996 to 2015)

**DOI:** 10.1186/s12885-023-10988-y

**Published:** 2023-06-19

**Authors:** Jew Win Kuan, Anselm Ting Su, Mastulu Wahab, Abdullah Hamdan, Jamilah Hashim, Andrew Kiyu, Choo Huck Ooi

**Affiliations:** 1 Department of Medicine, Faculty of Medicine and Health Sciences, Universiti Malaysia Sarawak (UNIMAS), 94300 Kota Samarahan, Sarawak Malaysia; 2 Department of Community Medicine and Public Health, Faculty of Medicine and Health Sciences, Universiti Malaysia Sarawak (UNIMAS), Kota Samarahan, Sarawak Malaysia; 3 Sarawak State Health Department, Ministry of Health, Kuching, Sarawak Malaysia

**Keywords:** Haematological cancer, Sarawak, Borneo, Registry, Acute leukaemia, Lymphoma, Myeloma, Plasma cell, Chronic myeloid leukaemia

## Abstract

**Background:**

Published epidemiological studies of haematological cancers are few. Hereby we present a 20-year epidemiological data of haematological cancers in Sarawak from a population-based cancer registry.

**Methods:**

Haematological cancer cases with ICD-10 coded C81-C96 and ICD-O coded /3 diagnosed from 1996 to 2015 were retrieved from Sarawak Cancer Registry. Adult was defined as those 15 years and above. Incidence rate (IR) was calculated based on yearly Sarawak citizen population stratified to age, gender, and ethnic groups. Age-standardised IR (ASR) was calculated using Segi World Standard Population.

**Results:**

A total of 3,947 cases were retrieved and analysed. ASR was 10 and male predominance (IR ratio 1.32, 95%CI 1.24,1.41). Haematological cancers generally had a U-shaped distribution with lowest IR at age 10–14 years and exponential increment from age 40 years onwards, except acute lymphoblastic leukaemia (ALL) with highest IR in paediatric 2.8 versus adult 0.5. There was a significant difference in ethnic and specific categories of haematological cancers, of which, in general, Bidayuh (IR ratio 1.13, 95%CI 1.00, 1.27) and Melanau (IR ratio 0.54, 95%CI 0.45, 0.65) had the highest and lowest ethnic-specific IR, respectively, in comparison to Malay. The ASR (non-Hodgkin lymphoma, acute myeloid leukaemia, ALL, chronic myeloid leukaemia, and plasma cell neoplasm) showed a decreasing trend over the 20 years, -2.09 in general, while Hodgkin lymphoma showed an increasing trend of + 2.80. There was crude rate difference between the 11 administrative divisions of Sarawak.

**Conclusions:**

This study provided the IR and ASR of haematological cancers in Sarawak for comparison to other regions of the world. Ethnic diversity in Sarawak resulted in significant differences in IR and ASR.

**Supplementary Information:**

The online version contains supplementary material available at 10.1186/s12885-023-10988-y.

## Background

Published epidemiological studies of haematological cancers are few, more so in Asia and Southeast Asia. Sarawak, one of the current three entities forming Malaysia, is located on Borneo Island, the third largest island in the world. Sarawak is the largest state in Malaysia with an area of 124,450 km^2^. Its population grew from1.86 million in 1996 to 2.54 million in 2015 and 2.91 million according to the latest census in 2020 [1]. It boasts 27 ethnic groups [2], of which the three major ethnic groups, as of the year 2010, are Iban (the biggest group of Dayak, i.e. Sarawak natives) (30.3%), Chinese (24.5%), and Malay (24.1%), followed by other Dayaks like Bidayuh (8.4%) and Melanau (5.2%) [3]. Each ethnic group has its own unique and distinct culture, language and lifestyle but all live in harmony. In 2012, Sarawak was divided into 11 administrative divisions consisting of administrative districts [3]. Thus, besides contributing to the epidemiological data of haematological cancers in Asia and Southeast Asia, an epidemiological study on haematological cancers in Sarawak might reveal an interesting and different discovery in view of the population diversity. At the same time, a study which includes 20-year cancer trend analysis would potentially trigger policy changes and provide learning points for other developing areas.

The Sarawak Cancer Registry (SCR) was set up by Sarawak State Health Department in 1996 as the regional population-based registry for patients with cancer in Sarawak. Over the last 30 years, SCR was committed to collect high quality cancer data in Sarawak through passive notification and active tracing (see MATERIALS AND METHODS). The data in SCR was previously published in periodic sessions, 1996 to 2000 [4], 2001 to 2005 [5], and the latest data 2005 to 2015 is in progress at the time of writing. As a complement to the SCR Report, this paper presents a more detailed analysis of the epidemiological data specifically on haematological cancer in Sarawak over a 20-year period, from year 1996 to 2015.

## Methods

### Source and completeness of data

The data was obtained from SCR managed by Sarawak State Health Department. The source of SCR was primarily based on passive voluntary notifications from all sections of the medical profession in Sarawak using a standardised format, called Cancer Notification Form (CNF) (Supplementary Data – Cancer Notification Form). In addition to that, active case finding and routine checks on government and private hospital discharge records and pathology record listings from the government and private laboratories were performed to ensure completeness of the data. This was supplemented by case detection from death records supplied by the National Registration Department. The cases picked up were then checked against data in SCR. If they were not yet notified, reminders were sent to the doctor-in-charge for more information. Additional missing data for these cases were sought by registry staff from the relevant hospital records.

Data were managed using the CanReg5 software from the International Association of Cancer Registries. The issue of multiple notifications of a patient was addressed by cross checking names and National Identification Card (IC) numbers, which are unique to each Malaysian citizen and permanent residents (PR), using the software.

This study was registered with the National Medical Research Register and approved by the Medical Research & Ethics Committee of the Ministry of Health, Malaysia (NMRR ID-22–01151-6KI (IIR)).

### Study and reference population

1. Study population was all cancer cases registered in the SCR, among citizens of Malaysia (which include PR of Malaysia and Sarawak) who resided, in Sarawak and whose cancers were diagnosed or treated in Sarawak.
2. Reference population no. 1 was Sarawak citizen population. The official Sarawak citizen population data, stratified by 5-year age group, gender, and ethnicity (only for the five major ethnic groups of Malay, Chinese, Iban, Bidayuh, and Melanau) for each year from 1996 to 2015, was obtained from Department of Statistics of Malaysia.
3. Reference population no. 2 was World Standard Population (Segi World Standard Population [6]) (Supplementary Data – World Standard Population (Segi World Standard Population)).
4. Reference population no. 3 was Sarawak population in 2016 [7] according to the 11 administrative divisions and gender. It was used for the calculation related to administrative divisions.

### Operational definition

- (1) Incidence Date, age, gender, and ethnicity were defined as the date of diagnosis stated in the CNF, the age from the date of birth or IC to Incidence Date, gender stated in the CNF, and ethnicity stated in the CNF, respectively.
- (2) Locality of the case was extracted from the usual residential address in CNF and coded according to the 11 administrative divisions mentioned in INTRODUCTION.
- (3) Diagnosis was manually coded according to International Statistical Classification of Diseases and Related Health Problems 10^th^ Revision (ICD-10) version 2019, while primary organ sites and morphology were manually coded according to the International Classification of Diseases for Oncology (ICD-O). For this study, all cases with ICD-10 coded C81-C96 (malignant neoplasms, stated or presumed to be primary, of lymphoid, haematopoietic and related tissue) and ICD-O coded /3 diagnosed from 1996 to 2015 were retrieved for further analysis.
- (4) Incidence was defined as the occurrence of new cases of disease (as defined by the diagnosis above) in a population over a specified period of time.
- (5) Crude Rate (CR) was defined as the sum of incidence observed in the study population in each year from 1996 to 2015, divided by the sum of the number of reference population no. 1 at risk in each year from 1996 to 2015 multiplied by 100,000.
  For CR according to administrative divisions, the denominator was derived from estimated population in the administrative division of the year based on the population distribution according to administrative divisions in 2016. For example, in Kuching Division,
  $${\mathrm{CR}}_{\mathrm{Kuching}1996} = {\mathrm{N}}_{\mathrm{Kuching}1996} / {\mathrm{N}}_{\mathrm{EstimatedPopulationKuching}1996} \times \mathrm{100,000}$$
  $${\mathrm{N}}_{\mathrm{EstimatedPopulationKuching}1996} = {\mathrm{N}}_{\mathrm{PopulationKuching}2016} / {\mathrm{N}}_{\mathrm{PopulationSarawak}2016} \times {\mathrm{N}}_{\mathrm{PopulationSarawak}1996}$$
- (6) Incidence Rate (IR) was used for age adjustment for comparative analysis across age groups since the incidence of cancer was mostly related to age. It was defined as the number of incidence in an age group in the study population during a defined period divided by the number of reference population no. 1 at risk in the same age group and period multiplied by 100,000. The IR, which is “age group-specific”, could be more specific i.e., gender- and/or ethnic-specific IR which was defined as the number of incidence in an age group in the specific gender and/or ethnic group of the study population during a defined period divided by the number of the specific gender and/or ethnic group of the reference population no. 1 at risk in the same age group and period multiplied by 100,000.
- (7) Age-standardised Incidence Rate (ASR) was used for age adjustment for comparative analysis since the incidence of cancer depends heavily on the age structure of the population. It was a summary measure, indicating the rate that a population would have if it had a standard age structure. It was calculated as follows:
  $$\mathrm{ASR} = \sum \left({\mathrm{IR}}_{\mathrm{i}} \times {\mathrm{w}}_{\mathrm{i}}\right) / \mathrm{total world standard population}$$
  IR_i_ - IRs in age group i; w_i_ - standard world population in age group i
- (8) Standard Error (SE) was used to show the amount of chance variation in CR and IR. It was calculated as follows:
  $$SE = \surd {\sum }_{i} {\mathrm{d}}_{\mathrm{i}} {\left({\mathrm{w}}_{\mathrm{i}}/{\mathrm{y}}_{\mathrm{i}}\right)}^{2}$$
  d_i_ - number of new cases of disease in age group i; w_i_ - standard world population in age group i; y_i_ - reference population no. 1 at risk in age group i
- (9) 95% Confidence Limit of ASR (95% CL) is endpoints of a range in which the true ASR would be expected to fall 95% of the time. It was calculated as follows:
  $$95\mathrm{\% CL }=\mathrm{ ASR }\pm \left(1.96 \times \mathrm{SE}\right)$$
- (10) Cumulative rate until completion of 74 years of age (CR74) was used to ascertain the cumulative risk and is expressed in percentages. It was calculated as follows:
  $$\mathrm{CR}74 = 5\mathrm{ x }{\sum }_{\mathrm{i}} \left({\mathrm{d}}_{\mathrm{i}}/{\mathrm{y}}_{\mathrm{i}}\right) \times 100$$
  d_i_ – number of new cases of disease in age group i; y_i_ – reference population no. 1 at risk in age group i
- (11) Cumulative Risk (CumR) is defined as a probability that an individual would develop cancer during a certain age period, in the absence of any competing cause of death. The age period over which the risk is accumulated in this study is 0 to 74 years. The precise mathematical relationship between the cumulative rate and the cumulative risk is:
- $$\mathrm{CumR}\;=\;100\;\times\;\left[1\;-\;\exp\;\left(-\mathrm{CR}74/100\right)\right]$$
  exp - exponential
- (12) Lifetime risk is defined as the likelihood that a person who is free of a certain type of cancer will develop or die from that type of cancer during his or her lifetime. Lifetime risk estimates are usually expressed as the odds of developing cancer (‘1 in x’) or as a percentage.
- (13) Specific haematological cancer categories were obtained from Diagnosis coded with ICD-10 and ICD-O in section (3) above (Supplementary Data – Supplementary Table 1). If there was discordance between ICD-10 and ICD-O, ICD-10 was amended based on ICD-O or data was amended based on original CNF. The list of the specific haematological cancer categories is shown in Supplementary Data – Supplementary Table 3. Of note, the category of myelodysplastic syndrome (MDS), an important haematological neoplasm was not included because it is not in ICD-10 coded C81-C96, and some diseases in the above categories were not included because they are not in ICD-10 coded C81-C96 and ICD-O coded /3.

### Statistical analysis

Descriptive analysis was performed using Excel for Microsoft 365. Multivariable analysis of incidence rate (c.f. Incidence Rate (IR)) was analysed with negative binomial regression analysis using Stata version 9.0 software (StataCorp, College Station, TX) and adjusted to gender, ethnic, and age groups. The result was reported as incidence rate ratio (IRR) and 95% confident interval (CI). The IRR is similar to risk ratio with the difference of including time variable in the calculation of incidence. The ratio compares the incidence of cancer in the target group of subjects against the reference group. Ratio of more than 1 indicates higher incidence in comparison to the reference group. The reference group used for the calculation of IRR are female, Malay, and age group of 0 to 4 year-old for gender, ethnic, and age groups respectively. Cancer trend analysis was conducted using Joinpoint Regression Program version 4.9.10 from the National Cancer Institute.

## Results

### Overall haematological cancers – incidence and CR

A total of 3,947 incidences were identified from SCR. CR of haematological cancers in Sarawak 1996 to 2015 was 8.9 per 100,000 population with male predominance against female (IRR 1.32, 95%CI 1.24,1.41) (Table 1). The lifetime risk of a male develops haematological cancer was 1.5 time higher than a female.

**Table 1:** Summary of haematological cancer incidence in Sarawak 1996 to 2015

Gender	Incidence	%	CR ^a^	SE	ASR ^a^	95% CL	CR74	CumR
Total	3,947	100.0	8.9	0.2	10.0	9.7, 10.3	1.0	1.0
Male	2,277	57.7	10.1	0.2	11.4	11, 11.9	1.2	1.2
Female	1,670	42.3	7.7	0.2	8.5	8.1, 9	0.8	0.8

*ASR* Age-standardised Incidence Rate, *CR* Crude Rate, *CR74* Cumulative rate until completion of 74 years of age, *CumR* Cumulative Risk, *SE* Standard Error, *95% CL* 95% Confidence Limit of ASR

^a^ per 100,000 population

Incidence in all the ethnic groups is shown in Supplementary Data – Supplementary Table 2. Bidayuh and Melanau had the highest and lowest CR among the five major ethnic groups, 10.9 and 5.3 per 100,000 population, respectively (missing data *n* = 4) (Table 2). There was a difference among the five major ethnic groups in comparison to Malay – Bidayuh and Melanau had the highest (IRR 1.13, 95%CI 1.00, 1.27) and lowest (IRR 0.54, 95%CI 0.45, 0.65) incidence rate, respectively. Iban also had a different incidence rate (IRR 0.86, 95%CI 0.79, 0.94) compared to Malay. There was no difference between Chinese and Malay (IRR 0.93, 95%CI 0.85, 1.01). Crude Rate gender ratio was also different among the five ethnic groups with Bidayuh had the highest male predominance, 1.4, while Melanau had the lowest, 1.1.

**Table 2:** Summary of haematological cancer incidence according to the major five ethnic groups in Sarawak 1996 to 2015

	**Incidence**			**CR **^**a**^				**SE**			**ASR**				**95% CL**			**CR74**		
	T	**M**	**F**	**T**	**M**	**F**	*R*	**T**	**M**	**F**	**T**	**M**	**F**	*R*	**T**	**M**	**F**	**T**	**M**	**F**
**Bidayuh**	**402**	239	163	**10.9**	12.8	8.9	***1.4***	**0.6**	1.0	0.8	**12.7**	15.2	10.1	***1.4***	**11.4, 13.9**	13.2, 17.2	8.5, 11.7	**1.4**	1.7	1.1
**Chinese**	**1,119**	635	484	**9.9**	10.9	8.8	***1.2***	**0.3**	0.5	0.4	**10.2**	11.3	9.0	***1.3***	**9.6, 10.8**	10.4, 12.2	8.2, 9.8	**1.0**	1.1	0.9
**Malay**	**929**	532	397	**8.9**	10.1	7.7	***1.3***	**0.4**	0.6	0.5	**10.8**	12.5	9.1	***1.4***	**10.0, 11.5**	11.3, 13.6	8.2, 10	**1.1**	1.3	0.9
**Iban**	**1,118**	651	467	**8.4**	9.7	7.1	***1.4***	**0.3**	0.4	0.4	**9.4**	10.9	7.9	***1.5***	**8.8, 9.9**	10.0, 11.7	7.2, 8.6	**1.0**	1.1	0.8
**Melanau**	**132**	70	62	**5.3**	5.5	5.0	***1.1***	**0.5**	0.7	0.7	**5.9**	6.1	5.6	***1.1***	**4.8, 6.9**	4.6, 7.5	4.2, 7.0	**0.6**	0.6	0.5

*ASR* Age-standardised Incidence Rate, *CR* Crude Rate, *CR74* Cumulative rate until completion of 74 years of age, *CumR* Cumulative Risk, *F* female, *M* male, *R* male to female ratio, *SE* Standard Error, *95% CL* 95% Confidence Limit of ASR

^a^ per 100,000 population

After excluding 255 missing data, a total of 3,692 incidences were analysed according to the 11 administrative divisions (Table 3). There was a difference in CR and CR gender ratio among the administrative divisions.

**Table 3:** Summary of haematological cancer incidence according to the 11 administrative divisions in Sarawak 1996 to 2015 (in descending order based on Crude Rate)

Division	Incidence			Incidence (%)			CR (per 100,000 population)			
	Total	M	F	Total	M	F	**Total**	M	F	***MFR***
**Samarahan**	402	240	162	10.2	10.5	9.7	**14.2**	8.7	5.5	***1.6***
**Sri Aman**	175	100	75	4.4	4.4	4.5	**10.4**	6.1	4.3	***1.4***
**Kuching**	1,295	744	551	32.8	32.7	33.0	**9.2**	5.4	3.8	***1.4***
**Betong**	169	98	71	4.3	4.3	4.3	**8.7**	5.3	3.5	***1.5***
**Sarikei**	181	100	81	4.6	4.4	4.9	**8.6**	4.9	3.7	***1.3***
**Sibu**	408	227	181	10.3	10.0	10.8	**7.6**	4.3	3.4	***1.3***
**Limbang**	106	64	42	2.7	2.8	2.5	**6.8**	4.1	2.7	***1.5***
**Kapit**	137	79	58	3.5	3.5	3.5	**6.7**	3.8	2.9	***1.3***
**Miri**	441	260	181	11.2	11.4	10.8	**6.7**	3.8	2.9	***1.3***
**Mukah**	128	72	56	3.2	3.2	3.4	**6.3**	3.5	2.8	***1.2***
**Bintulu**	250	143	107	6.3	6.3	6.4	**6.2**	3.3	2.8	***1.2***

*CR* Crude Rate, *F* female, *M* male, *MFR* male to female ratio

### Overall haematological cancers – IR, gender- and/or ethnic-specific IR, and ASR

**Fig. 1:**
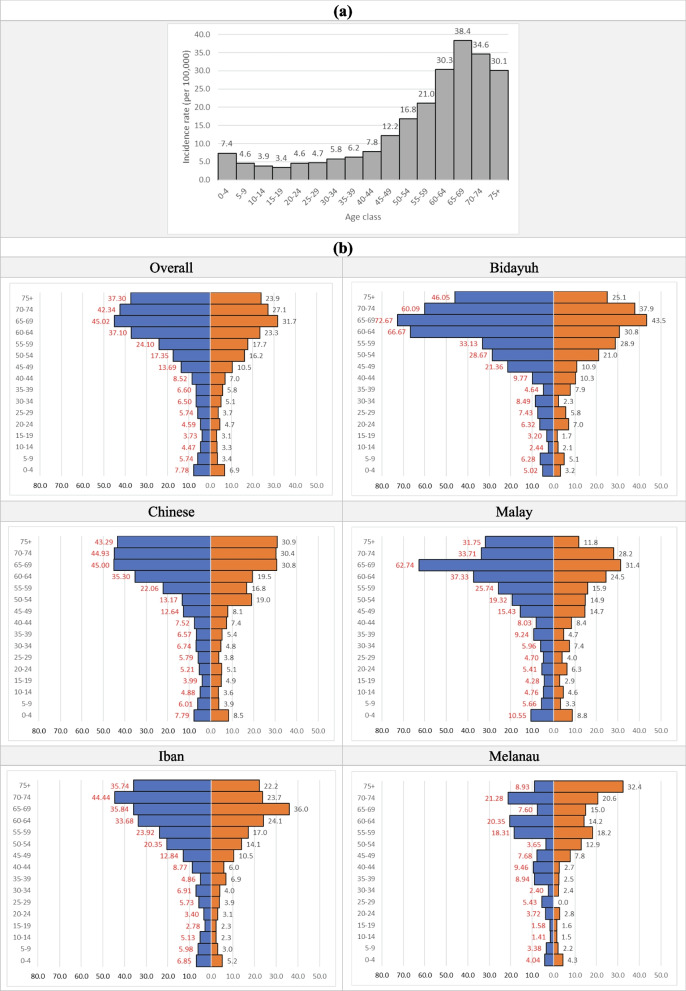
**a** The Incidence Rate of haematological cancers in Sarawak 1996 – 2015. **b** The gender- and ethnic-specific Incidence Rate of haematological cancers in Sarawak 1996 – 2015 for overall and the five major ethnic groups. Y-axis denotes age groups and X-axis denotes gender- and ethnic-specific Incidence Rate (per 100,000 population). The unit of the X- and Y-axis are the same for all the figures above for ease of comparison. Blue and red bar represent male and female, respectively

Incidence Rate showed a peak at initial years of life (age 0 to 4) which reduced to the lowest at the age of 15 to 19 years, then slowly increased with age with exponential rise after the age of 40 years for both genders till the age of 65–69 years. The average of gender-specific IR male to female ratio was 1.3 with higher ratio of 1.6 in older age group of 60 years and above (Fig. 1).

The pattern of distribution of the gender- and ethnic-specific IR among the five major ethnic groups was similar both to each other and to that of the whole population (Fig. 1). In older age group of 60 years and above, gender-specific IR male to female ratio still showed male predominance in all four ethnic groups with the highest in Malay, 1.9, followed by Bidayuh, 1.8, while Melanau had an inverse ratio, 0.8.

As the population distribution across the 5-year age groups might be different among the five major ethnic groups, ASR is a better representation than CR which could be appreciated in Table 2. The descending order of ASR according to the ethnic groups was Bidayuh 12.7 per 100,000 population, Malay 10.8, Chinese 10.2, Iban 9.4, and Melanau 5.9. The highest ASR gender ratio was also different from CR gender ratio, of which Iban was the highest, 1.5, while the lowest was the same, Melanau, 1.1.

### Specific haematological cancer categories

Incidence of specific haematological cancer categories is shown in Supplementary Data – Supplementary Table 3. Among the three major haematological cancer categories, namely lymphoma, acute leukaemia (AL), and plasma cell neoplasm (PCN), or more commonly known as lymphoma, leukaemia, and myeloma, the CR ratio was 8: 4.5: 1 (Table 4). Crude Rate of non-Hodgkin lymphoma (NHL)/leukaemia was 8 times higher than Hodgkin lymphoma (HL). Among NHL/leukaemia, CR of mature B cell was 7.5 times higher than T cell. Crude Rate of acute myeloid leukaemia and related precursor neoplasms (AML) which includes acute promyelocytic leukaemia (APML) was similar to precursor lymphoid neoplasms i.e., acute lymphoblastic leukaemia (ALL). Of note, the incidence of mature B cell NHL/leukaemia only began to increase since 2009 because immunohistochemical staining of sample has only become more widely available since then. Male predominance was present in all haematological cancer categories except AML and mature T cell NHL/leukaemia which had equal gender CR.

**Table 4:** Crude Rate of various haematological cancer categories in Sarawak 1996 to 2015 (per 100,000 population)

Disease category	All				Paediatric				Adult			
	**T**	M	F	***R***	**T**	M	F	***R***	**T**	M	F	***R***
**Lymphoma**	**4.9**	5.7	4.1	***1.4***	**1.0**	1.4	0.7	***2.1***	**6.8**	7.8	5.7	***1.4***
**AL**	**2.7**	2.9	2.6	***1.1***	**4.0**	4.4	3.7	***1.2***	**2.1**	2.2	2.0	***1.1***
**PCN**	**0.6**	0.8	0.5	***1.5***	**0.0**	-	-	*-*	**0.9**	1.1	0.7	***1.5***
**NHL/leukaemia**	**4.3**	5.0	3.6	***1.4***	**0.8**	1.1	0.5	***2.1***	**6.0**	6.9	5.0	***1.4***
**HL**	**0.6**	0.7	0.5	***1.5***	**0.2**	0.3	0.1	***2.1***	**0.8**	1.0	0.7	***1.4***
**Mature B cell NHL/leukaemia**	**1.3**	1.4	1.1	***1.4***	**0.2**	0.3	0.2	***1.8***	**1.7**	2.0	1.5	***1.3***
**Mature T cell NHL/leukaemia**	**0.2**	0.2	0.2	***1.0***	**0.1**	0.1	0.1	***1.0***	**0.2**	0.2	0.2	***1.1***
**AML**	**1.3**	1.4	1.3	***1.0***	**1.1**	1.2	1.0	***1.3***	**1.5**	1.4	1.5	***1.0***
**ALL**	**1.2**	1.4	1.1	***1.3***	**2.8**	3.1	2.5	***1.2***	**0.5**	0.6	0.4	***1.5***

*F* female, *M* male, *R* male CR to female CR ratio; *T* total

As the IR was lowest at the age group of 15 to 19 years (Fig. 1) and there was no standard definition of paediatric and adult ages, the cut-off age of 15 years was used to compare between paediatric and adult haematological cancers. Ages of 15 years and above was categorised as adult, while below as paediatric. There was a difference in CR ratio of the above specific haematological cancer categories between paediatric and adult (Table 4). In paediatric, PCN was extremely rare and CR ratio of lymphoma: AL was 1: 4. Crude Rate ratio of NHL: HL was 4: 1, mature B cell NHL: T cell NHL/leukaemia was 4: 1, and AML: ALL was 1: 2.5. In adult, CR ratio of lymphoma: AL: PCN was 7.5: 2.5: 1, NHL: HL was 7.5: 1, mature B cell NHL: T cell NHL/leukaemia was 8: 1, and AML: ALL was 3: 1. Haematological cancers were generally male predominant in both paediatric and adult, but certain cancer categories had higher male predominance in paediatric than adult i.e., HL, 2.1 versus 1.4, and mature B cell NHL/leukaemia, 1.8 versus 1.3.

**Fig. 2:**
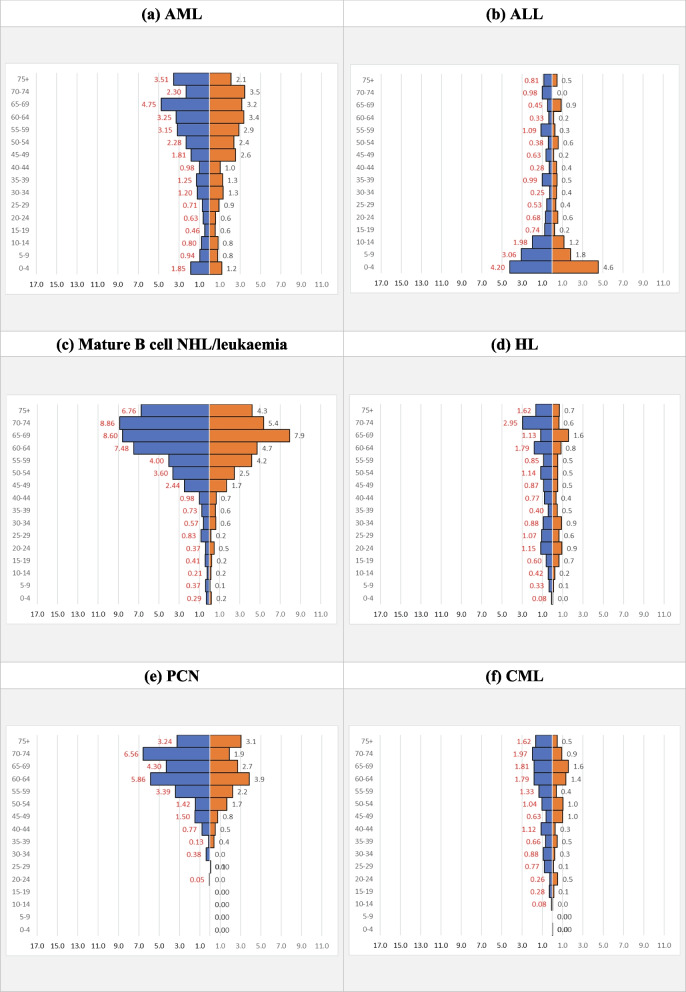
The gender-specific Incidence Rate of haematological cancers in Sarawak 1996 – 2015 for the six selected haematological cancers. Y-axis denotes age groups and X-axis denotes gender-specific Incidence Rate (per 100,000 population). The unit of the X- and Y-axis are the same for all the figures above for ease of comparison. Blue and red bar represent male and female, respectively

**Fig. 3:**
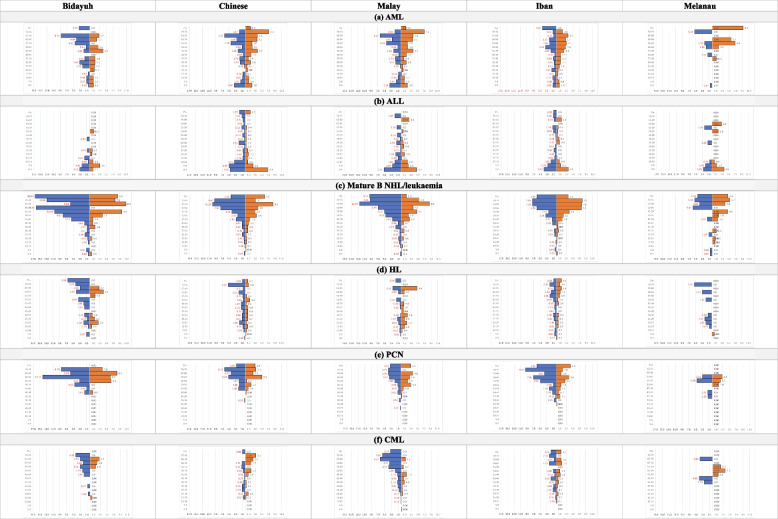
The gender- and ethnic-specific Incidence Rate of haematological cancers in Sarawak 1996 – 2015 for the five major ethnic groups and the six selected haematological cancers. Y-axis denotes age groups and X-axis denotes gender- and ethnic-specific Incidence Rate (per 100,000 population). The unit of the X- and Y-axis are the same for all the figures above and Fig. 2 for ease of comparison. Blue and red bar represent male and female, respectively

AML, ALL, mature B cell NHL/leukaemia, HL, PCN, and chronic myeloid leukaemia (CML) were further analysed because the total incidence was more than 200. Summary of the incidence and CR of the six selected haematological cancers is shown in Supplementary Data – Supplementary Table 4. The pattern of gender-specific IR in AML, mature B cell NHL/leukaemia and PCN were similar, showing an inverted pyramidal shape with IR increased exponentially from the age of 40 (Fig. 2). On the other hand, ALL showed a pyramidal shape, with a linear decreasing trend across age groups between 0 to 19. IR showed a slow increasing trend across age groups in HL and CML. Incidence Rate of the six selected cancers was further breakdown according to the five major ethnic groups as shown in Fig. 3.

All the six selected cancers were male predominant, except AML which had an equal ASR gender ratio (Table 5). The highest male predominance was in CML, 1.6, and lowest in ALL, 1.3. The commonest cancer in male and female was mature B cell NHL/leukaemia, 1.7, and AML, 1.4, respectively.

**Table 5:** ASR for the six selected haematological cancers in Sarawak 1996 to 2015 (per 100,000 population)

		**All**				**Bidayuh**				**Chinese**				**Malay**				**Iban**				**Melanau**			
		**T**	M	F	***R***	**T**	M	F	***R***	**T**	M	F	***R***	**T**	M	F	***R***	**T**	M	F	***R***	**T**	M	F	***R***
**AML**	Paed	**0.3**	0.4	0.3	***1.3***	**0.3**	0.2	0.3	***0.8***	**0.3**	0.3	0.3	***0.8***	**0.5**	0.6	0.4	***1.4***	**0.3**	0.3	0.2	***1.4***	**0.0**	0.1	0.0	
	Adult	**1.1**	1.1	1.1	***1.0***	**1.1**	1.3	0.9	***1.4***	**1.3**	1.4	1.2	***1.1***	**1.1**	1.0	1.2	***0.9***	**1.0**	0.9	1.0	***0.8***	**0.6**	0.4	0.9	***0.4***
	**Total**	**1.5**	1.5	1.4	***1.0***	**1.4**	1.6	1.3	***1.3***	**1.6**	1.6	1.6	***1.0***	**1.6**	1.6	1.6	***1.0***	**1.2**	1.2	1.3	***0.9***	**0.7**	0.5	0.9	***0.5***
**ALL**	Paed	**0.9**	1.0	0.8	***1.2***	**0.6**	0.6	0.5	***1.2***	**1.2**	1.3	1.1	***1.2***	**1.0**	1.0	0.9	***1.1***	**0.8**	0.9	0.6	***1.5***	**0.6**	0.6	0.6	***0.9***
	Adult	**0.3**	0.4	0.3	***1.5***	**0.2**	0.3	0.2	***1.5***	**0.4**	0.5	0.3	***1.6***	**0.4**	0.5	0.3	***1.6***	**0.3**	0.4	0.2	***1.8***	**0.2**	0.2	0.2	***0.8***
	**Total**	**1.3**	1.4	1.1	***1.3***	**0.8**	0.9	0.7	***1.3***	**1.7**	1.9	1.4	***1.3***	**1.4**	1.6	1.3	***1.2***	**1.1**	1.3	0.8	***1.6***	**0.8**	0.8	0.9	***0.9***
**Mature B cell NHL/ leukaemia**	Paed	**0.1**	0.1	0.1	***1.8***	**0.1**	0.2	0.1	***2.5***	**0.0**	0.1	0.0	***1.7***	**0.1**	0.1	0.1	***1.0***	**0.1**	0.1	0.0	***2.0***	**0.1**	0.1	0.0	
	Adult	**1.4**	1.6	1.2	***1.3***	**2.2**	3.0	1.5	***2.0***	**1.4**	1.6	1.2	***1.3***	**1.5**	2.0	1.1	***1.8***	**1.3**	1.2	1.3	***1.0***	**0.8**	0.7	0.9	***0.8***
	**Total**	**1.5**	1.7	1.3	***1.4***	**2.3**	3.1	1.5	***2.0***	**1.5**	1.7	1.2	***1.4***	**1.6**	2.0	1.2	***1.7***	**1.3**	1.3	1.3	***1.0***	**0.9**	0.9	0.9	***1.0***
**HL**	Paed	**0.1**	0.1	0.0	***2.1***	**0.0**	0.1	0.0		**0.1**	0.1	0.1	***1.3***	**0.0**	0.1	0.0	***2.0***	**0.1**	0.1	0.0	***3.0***	**0.0**	0.0	0.1	***0.0***
	Adult	**0.6**	0.7	0.5	***1.5***	**1.0**	1.2	0.7	***1.7***	**0.6**	0.8	0.4	***1.9***	**0.6**	0.5	0.7	***0.7***	**0.5**	0.6	0.4	***1.5***	**0.4**	0.8	0.0	
	**Total**	**0.6**	0.8	0.5	***1.5***	**1.0**	1.3	0.7	***1.8***	**0.7**	0.9	0.5	***1.8***	**0.6**	0.5	0.7	***0.7***	**0.6**	0.7	0.4	***1.7***	**0.4**	0.8	0.1	***10.7***
**PCN**	Paed	**0.0**	0.0	0.0		**0.0**	0.0	0.0		**0.0**	0.0	0.0		**0.0**	0.0	0.0		**0.0**	0.0	0.0		**0.0**	0.0	0.0	
	Adult	**0.8**	0.9	0.6	***1.5***	**1.1**	1.4	0.9	***1.6***	**0.7**	0.8	0.6	***1.4***	**0.6**	0.7	0.6	***1.1***	**0.9**	1.2	0.7	***1.7***	**0.4**	0.5	0.3	***1.5***
	**Total**	**0.8**	0.9	0.6	***1.5***	**1.1**	1.4	0.9	***1.6***	**0.7**	0.8	0.6	***1.4***	**0.6**	0.7	0.6	***1.1***	**0.9**	1.2	0.7	***1.7***	**0.4**	0.5	0.3	***1.5***
**CML**	Paed	**0.0**	0.0	0.0	***0.8***	**0.0**	0.0	0.0		**0.0**	0.0	0.0		**0.0**	0.0	0.0	***0.8***	**0.0**	0.0	0.0	***1.0***	**0.0**	0.0	0.0	
	Adult	**0.5**	0.6	0.4	***1.6***	**0.5**	0.7	0.3	***2.5***	**0.4**	0.4	0.5	***0.9***	**0.7**	1.1	0.3	***3.4***	**0.5**	0.6	0.4	***1.3***	**0.4**	0.5	0.4	***1.4***
	**Total**	**0.5**	0.6	0.4	***1.6***	**0.5**	0.7	0.3	***2.5***	**0.4**	0.4	0.5	***0.9***	**0.7**	1.1	0.3	***3.2***	**0.5**	0.6	0.4	***1.3***	**0.4**	0.5	0.4	***1.4***

*F* female, *M* male, *R* male to female ratio, *T* total

There were differences in ASR of the six selected cancers among the five major ethnic groups (Table 5). In general, Melanau had the lowest ASR for all the six selected cancers, between 0.4 and 0.9 per 100,000 population. Bidayuh had the highest ASR for mature B cell NHL/leukaemia, HL, and PCN, 2.3, 1.0, and 1.1, respectively, compared to the lowest in Melanau, 0.9, 0.4, and 0.4, respectively. Chinese and Malay had the highest ASR for AML, 1.6, compared to the lowest 0.7 in Melanau. Chinese had the highest ASR for ALL, 1.7, while Bidayuh and Melanau had the lowest, 0.8. Malay had the highest CR for CML, 0.7, compared to the lowest, 0.4, in Melanau and Chinese. The ASR gender ratios were also different among the five major ethnic groups, but generally still male predominant. The highest ASR gender ratio was 10.7 in HL Melanau, while the lowest was 0.5 in AML Melanau. The loss of male predominance was seen more in Melanau, while Bidayuh showed male predominance in all the six selected cancers. Chinese showed loss of male predominance in AML and CML, Malay in AML and HL, and Iban in AML and mature B cell NHL/leukaemia.

In Bidayuh, the commonest among the six selected cancers was mature B cell NHL/leukaemia with ASR of 2.3, followed by AML, 1.4, while the rarest was CML, 0.5 (Table 5). In Chinese, the commonest was ALL, 1.7, followed by AML, 1.6, while the rarest was CML, 0.4. In Malay, the commonest was mature B cell NHL/leukaemia and AML, 1.6, followed by ALL, 1.4, while the rarest was HL and PCN, 0.6. In Iban, the commonest was mature B cell NHL/leukaemia, 1.3, followed by AML, 1.2, while the rarest was CML, 0.5. In Melanau, the commonest was mature B cell NHL/leukaemia, 0.9, followed by ALL, 0.8, while the rarest was HL, PCN and CML, 0.4.

In paediatric, the ASR gender ratio was generally more than adult except in ALL, 1.2 versus 1.5 (Table 5). The highest ASR gender ratio in paediatric was seen in HL, 2.1 with Iban had the highest, 3.0. ALL is the commonest haematological cancer in paediatric. However, there was an ethnic difference with the highest in Chinese, 1.2, followed by Malay, 1.0, while the lowest in Bidayuh and Melanau, 0.6. It appeared that Malay paediatric had a higher risk of getting AML as compared to other ethnic groups. It seems like Chinese had more haematological cancer involving lymphoid lineage during childhood but myeloid lineage disease increases in adulthood, while the other four ethnic groups continued to have more lymphoid lineage disease in adulthood.

In adult, the highest ASR gender ratio was 3.4 in CML Malay and lowest 0.4 in AML Melanau (Table 5).

From the available subtyping data, the two major subtypes of HL were nodular sclerosis (ICD-10: C811; ICD-O: 9663/3 – 9667/3) 29/52 (55.8%) and mixed cellularity (ICD-10: C812; ICD-O: 9652/3) 19 (36.5%).

The three major subtypes of mature B cell NHL/leukaemia were diffuse large B cell lymphoma (ICD-10: C833; ICD-O: 9680/3, 9684/3, 9688/3, 9735/3, 9737/3) 346/546 (63.4%), chronic lymphocytic leukaemia/small lymphocytic lymphoma (CLL/SLL) (ICD-10 and ICD-O for CLL: C911 and 9823/3; for SLL: nil (no specific coding) and 9670/3) 78 (14.3%), and follicular lymphoma (ICD-10: C820 – C829; ICD-O: 9695/3, 9691/3, 9698/3, 9597/3, 9675/3, 9690/3) 66 (12.1%).

The two major subtypes of mature T cell NHL/leukaemia were peripheral T-cell lymphoma, not elsewhere classified (ICD-10: C844; ICD-O: 9720/3) 31/75 (41.3%) and anaplastic large cell lymphoma, ALK-positive (ICD-10: C846; ICD-O: 9714/3) 23 (30.7%).

Among the specific subtypes of AML, APML (ICD-10: C924; ICD-O: 98,660/3) consisted of 30/596 (5.0%), acute erythroid leukaemia (ICD-10: C940; ICD-O: 9840/3) 5 (0.8%), and acute megakaryoblastic leukaemia (ICD-10: C942; ICD-O: 9910/3, 9911/3) 2 (0.3%). Among ALL, B cell (ICD-10: nil; ICD-O: 9836/3) was 27/34 (79%) and T cell (ICD-10: nil; ICD-O: 9837/3) was 7 (21%).

#### Trend analysis

**Fig. 4:**
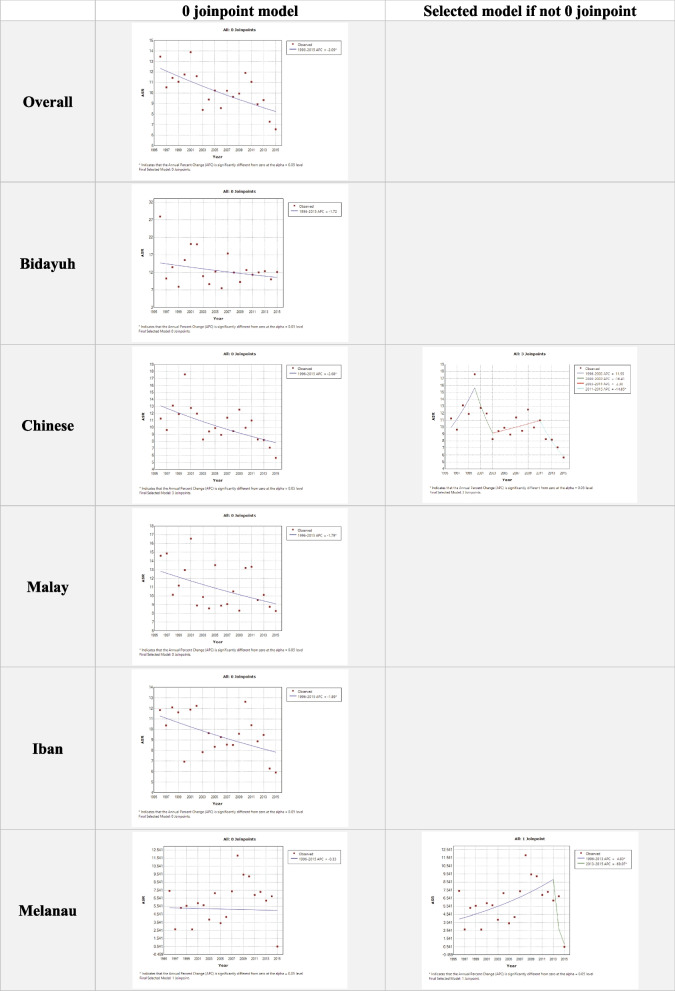
The ASR trend of haematological cancers in Sarawak 1996 – 2015 for overall population and five major ethnic groups

From 1996 to 2015, the ASR of haematological cancers in Sarawak showed a decreasing trend at -2.09 (Fig. 4). ASR among all the major ethnic groups showed a decreasing trend ranging from -1.72 to -2.68, except for Melanau which showed an increasing trend of + 4.83.

**Fig. 5:**
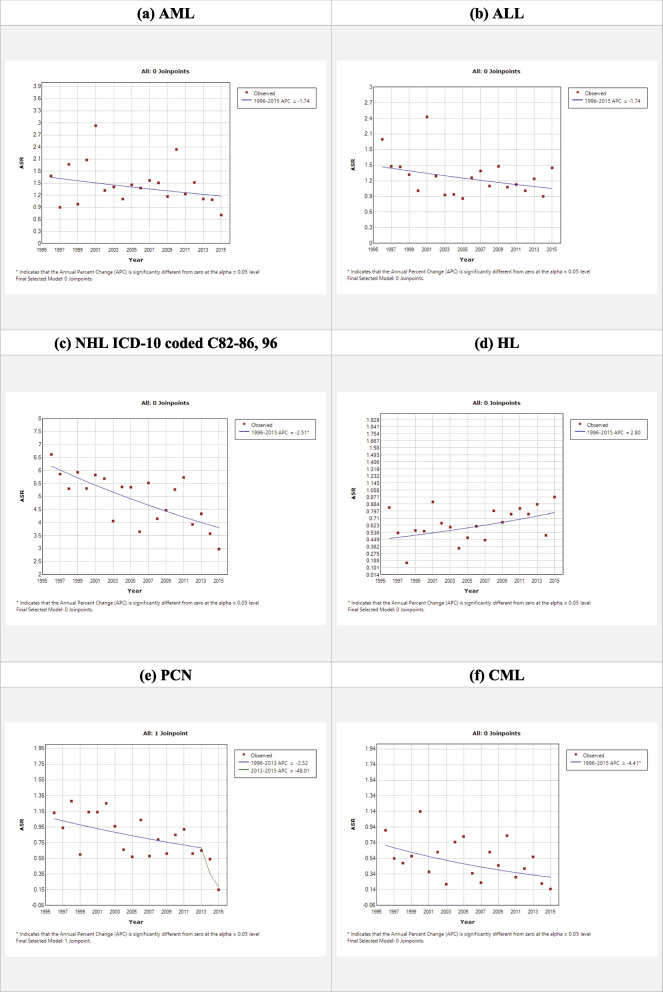
The ASR trend of the six selected haematological cancers in Sarawak 1996 – 2015

For the six selected haematological cancers (mature B cell NHL/leukaemia was replaced with NHL ICD-10 coded C82-86,96), all showed a decreasing ASR trend ranging from -1.74 to -4.41, except HL which showed an increasing trend of + 2.80 (Fig. 5).

**Fig. 6:**
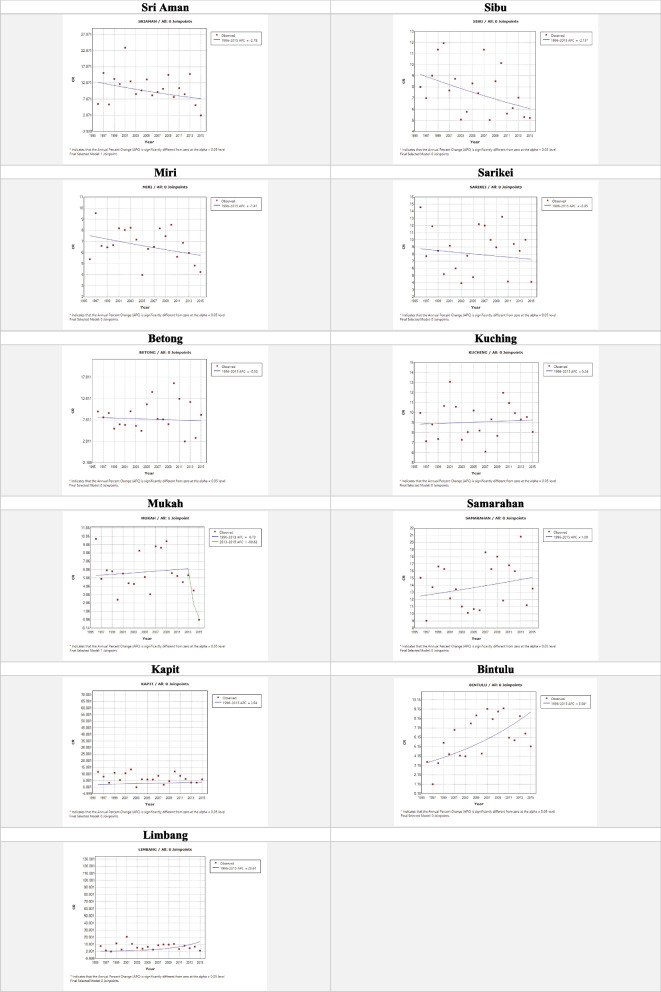
The CR trend of the 11 administrative divisions in Sarawak 1996 – 2015 in ascending order

The CR trend of haematological cancers in the 11 administrative divisions is shown in Fig. 6. Three administrative divisions, Sri Aman, Sibu, and Miri showed a decreasing ASR, of which Sri Aman had the highest decreasing ASR trend of -2.78. Three divisions, Sarikei, Betong, and Kuching, had a similar ASR of ± 0.9 over the 20 years. Four divisions, Samarahan, Kapit, Bintulu, and Limbang, showed an increasing ASR trend, of which Limbang had the highest ASR trend of 20.61.

## Discussion

This is the first epidemiology study on haematological cancers in Malaysia. It provided data in general and on some aspects more relevant to daily clinical haematological practice and highlighted the differences unique to the diverse Sarawak population.

### Some unexpected findings

- (1) The decreasing trend of ASR in overall, and in AML, ALL, CML, and PCN (Fig. 5) might be due to improvement of socioeconomic and health status. The comparison with other countries is discussed below.
- (2) The differences among ethnic groups were expected, but the magnitude of the differences was unexpected. It is known that the three indigenous Dayak populations in Sarawak (Iban, Bidayuh, and Melanau) are distinctly different from one another, and the three major ethnic groups in Malaysia (Malay, Chinese and Indian). Based on the result of haplotypes and allele frequencies of 17 Y-chromosomal short tandem repeat (Y-STR), Melanau had a strikingly high degree of shared haplotypes within [8]. The underlying genetic difference among the ethnic groups are likely amplified further by external factors i.e. environment, lifestyle, and diet [9]. Bidayuh had the highest ASR of haematological cancer, 12.7 per 100,000 population (Table 2), notably in mature B cell NHL/leukaemia, HL, and PCN (Table 5). Similarly, Bidayuh was also reported to have the highest incidence rate of nasopharyngeal carcinoma compared to other ethnic groups in Sarawak and other parts of the world [10]. On the other hand, Melanau had the lowest ASR of haematological cancers, 5.9 (Table 2), and the six selected haematological cancers (Table 5). At the time of writing, the analysis of other cancers among the five ethnic groups was still in progress, and it would be interesting to explore these findings.
  The gender difference among ethnic groups was also seen. Bidayuh had the highest CR gender ratio of 1.4 (Table 2) and went as high as 2.7 in CML (data not shown). The ratio was also around 2.7 in nasopharyngeal carcinoma [10]. Melanau, again, had the lowest ratio of 1.1, but unusually high ratio of 8.8 in HL and reverse ratio of 0.5 in AML.
  The differences among ethnic groups mentioned above warrant further studies and exploration.
- (3) Kuching is the capital city of Sarawak and was resided by 30% of the Sarawak population [7]. However, the highest CR was not in Kuching, but Samarahan and Sri Aman (Table 3). We are unsure of the reason. Further study is warranted.

### Comparison with other countries

- (1) The ASR of specific haematological cancer categories in Sarawak in comparison to other countries/continents [11] is shown in Table 6. In general, the ASR was in a lower range in comparison to other countries/continents.
- (2) The ASR trend of specific haematological cancer categories (Fig. 5).

**Table 6:** ASR of specific haematological cancer categories in Sarawak 2008 to 2012 and other countries/continents 2008 to 2012

Country/Continent	HL (C81)		NHL (C82-86, C96)		Multiple myeloma (C90)	
	Male	Female	Male	Female	Male	Female
**Sarawak – all ethnic**	0.9	0.7	5.3	4.0	0.8	0.7
**Sarawak – Malay**	0.7	0.8	5.9	4.6	0.3	0.5
**Sarawak – Chinese**	0.7	0.9	4.4	3.5	0.8	0.7
**Sarawak – Iban**	1.0	0.4	5.6	3.8	1.1	0.9
**Sarawak – Bidayuh**	1.3	1.1	6.8	6.2	0.6	1.5
**Sarawak – Melanau**	0.6	0.0	4.1	3.6	1.1	0.3
**Malaysia (Penang) – all ethnic**	0.9	0.5	7.4	5.5	0.8	0.7
**Malaysia (Penang) – Malay**	1.1	0.6	6.6	5.8	0.8	0.7
**Malaysia (Penang) – Chinese**	0.7	0.4	7.6	5.8	0.9	0.6
**Singapore**	NA	NA	NA	NA	NA	NA
**Thailand**	0.1 to 0.9	0.2 to 0.5	5.6 to 9.2	3.7 to 6.7	0.9 to 1.2	0.8 to 1.2
**Indonesia**	NA	NA	NA	NA	NA	NA
**China**	0.1 to 0.9	0.1 to 0.6	1.8 to 7.6	1.1 to 5.4	0.3 to 4.5	0.1 to 0.3
**Korea (South)**	0.3 to 0.7	0.2 to 0.4	6.5	4.1	1.8	1.2
**Japan**	0.6 to 1.2	0.4 to 0.7	7.9 to 10.0	5.8 to 6.8	1.6 to 2.1	1.2 to 1.6
**Western Europe: UK**	2.9	2.2	12.4	9.0	4.6	3.0
**Eastern Europe: Russian**	1.6 to 2.8	1.5 to 2.6	3.9 to 5.5	3.2 to 4.0	1.0 to 1.7	1.3 to 1.6
**North America: USA**	2.7	2.2	14.1	9.7	4.7	3.2
**South America: Brazil**	1.2 to 3.1	1.4 to 2.2	6.4 to 15.0	5.2 to 9.9	2.4 to 5.1	1.9 to 4.2
**Africa**	0.4 to 1.8	0.0 to 2.3	1.6 to 16.1	1.1 to 11.8	0.5 to 3.7	0.2 to 4.0
**Oceania: Australia**	2.7	2.1	15.3	10.3	4.9	3.2

Of note, the discussion in this sub-section should be interpreted together with the limitation of the study discussed in the sub-section below.

The estimated average percentage change in Sarawak from 1996 to 2015 for AML, ALL, CML, and CLL in comparison with other countries [12] is shown in Table 7.

**Table 7:** Estimated average percentage change in Sarawak 1996 to 2015 and other countries/continents 1990 to 2017

Country/Continent	ALL	CLL	AML	CML
Sarawak	-1.74	^a^	-1.74	-4.41
Malaysia	-0.66	2.83	0.56	0.06
Singapore	-2.61	2.97	0.31	-2.82
Thailand	-0.21	3.00	1.17	-0.51
Indonesia	1.36	2.58	1.22	0.35
China	1.31	5.99	1.54	-1.06
Korea (South)	-2.31	5.54	-0.04	-0.61
Japan	-2.16	2.09	1.08	-3.62
Western Europe	-2.14	0.14	0.40	-4.12
Eastern Europe	-1.60	2.50	-0.59	-0.55
North America	-1.47	-0.16	1.04	-4.28
South America	-0.23	0.18	0.18	-3.29
Africa	-1.36 to 0.36	0.39 to 1.88	-0.26 to 0.62	-1.41 to 0.58
Oceania	-0.21	0.37	0.23	-1.06

*EAPC* estimated average percentage change

^a^ Could not analyse the cohort because ASR was 0 in 1999 and 2000; ASR ranged from 0 to 0.5

The decreasing trend of ASR in AML was contradicting with the increasing trend observed in most countries between 1990 and 2017 [12].

For ALL, the same decreasing trend was observed in most countries between 1990 and 2017 [12]. Interestingly, the decreasing trend in South Korea [12] is contradicting with another study which showed an increasing trend in lymphoid leukaemia between 1999 to 2018 [13]. This might be due to increasing trend of lymphoid leukaemia other than ALL. However, the decreasing trend in Spain [12] is consistent with another study between 1995 to 2015 [14].

For CML, the CR of 0.5 per 100,000 population in Sarawak between 1995 to 2015 was similar to the previously reported 0.8 in southern Sarawak between 2011 to 2016 [15]. The decreasing trend was observed in most countries between 1990 and 2017 [12]. The decreasing trend in South Korea [12] is, again, not shown by another study which showed an increasing trend in myeloid leukaemia between 1999 to 2018 [13]. This is most likely masked by the increasing trend of AML, a much commoner myeloid leukaemia than CML.

PCN is a haematological cancer category with a known high incidence rate in elderly, of which the same observation was seen in Sarawak population among those aged 60 years and above (Fig. 2 (e)). With the aging population and better sociodemographic index, the decreasing trend in PCN was contradicting with the expected increment seen in other studies using global data from 1990 to 2016 [16].

For NHL with ICD-10 coded C82-86, the increasing ASR trend in Sarawak 1996 to 2015 was consistent with most countries [17].

### Limitations of the study

Like most registry data, this study is also limited by under-reporting. The active case finding and routine checks had reduced the under-reporting rate, but a significant number was likely persisted as evidenced by only 4 incidence of PCN in 2015 which is very unlikely based on the authors’ clinical experience and wave-like pattern in the overall incidence over the 20 years (Fig. 4). To our postulation, the under-reporting could be due to, firstly, a limited access to health care system in the remote areas in Sarawak between 1996 and 2015 that led to under-diagnosed cases. However, this would reduce as Sarawak develops. Secondly, the data source in the study did not include death record registry to capture unnotified cases that might escape active case finding and routine checks. Thirdly, changes in personnel in the system might led to inconsistent workflow in diagnosing, notifying, and finding/checking. However, this could be reduced by including data over a longer period of time.

MDS and some diseases in the specific haematological cancer categories (Supplementary Data – Supplementary Table 3) are important haematological neoplasms which contribute significant workload to the daily clinical haematological practice. However, they were not included in the analysis (see MATERIALS AND METHODS) because they are likely under-diagnosed and under-reported. For example, to diagnose MDS in the presence of cytopaenia, an invasive procedure involving bone marrow aspiration and trephine biopsy is required to assess for dysplasia and genetic abnormalities. However, in an asymptomatic elderly patient with mild cytopaenia not caused by other identifiable causes, the procedure might be refused or delayed by the treating physician and/or patient who opted for blood count monitoring only. Thus, this study was confined to haematological cancers with ICD-10 coded C81-C96.

There are many classifications of haematological neoplasms, which evolve and/or merge over time as our understanding of these diseases advances. The World Health Organization (WHO) Classification of Tumours of Haematopoietic and Lymphoid Tissues is the reference classification used in daily clinical practice, and it is updated every few years. The latest version is the recently released 5^th^ edition [18, 19]. The evolution of the classification of haematological neoplasms must be considered during data interpretation of the study. Firstly, the study was unable to classify 1,277 cases of NHL/lymphoid leukaemia, not otherwise specified (NOS), 11 cases of myeloid leukaemia, NOS, and 46 cases of leukaemia, NOS (Supplementary Data – Supplementary Table 3) into more specific categories/diseases due to the evolution of the classification of haematological neoplasms over time and logistic constraint. Secondly, the incidence of mature B or T cell NHL/leukaemia was unavailable prior to 2009 because immunohistochemical staining of samples has only become more widely available in Sarawak since then. Thirdly, the study could not provide information on certain important specific subtypes for daily clinical haematological practice like ALL with *BCR::ABL1* fusion, and AML with *RUNX1::RUNX1T1* fusion and *CBFB::MYH11* fusion. AML with *CBFB::MYH11* fusion is not specified in both ICD-10 and ICD-O, while ALL with *BCR::ABL1* fusion and AML with *RUNX1::RUNX1T1* fusion are not specified in ICD-10 but specified in ICD-O, code: 9812/3 and 9896/3, respectively. There was no cases of ALL with *BCR::ABL1* fusion and 15 cases of AML with *RUNX1::RUNX1T1* fusion in the study which does not correspond to common epidemiology and authors’ clinical experience. Regardless of specification in ICD-10 or ICD-O, specific subtypes were likely under-reported because result of the specific genetic tests are usually delayed and unavailable at the time of diagnosis and reporting of ALL or AML.

## Conclusions

This study provided useful information on various epidemiological aspects of haematological cancers in Sarawak between 1996 and 2015 based on a high-quality data extracted from SCR with annotation on certain limitations. The ASR was 10 per 100,000 population with a male predominance of approximately 57%. CML and AML had the highest and lowest ASR gender ratio, 1.6 and 1.0, respectively. There was a significant difference among the five major ethnic groups, of which, in general, Bidayuh and Melanau group had the highest and lowest ASR, 12.7 and 5.9, respectively. The CR ratio of lymphoma: AL was 1: 4 in paediatric and lymphoma: AL: PCN was 7.5: 2.5: 1 in adult. The ASR showed a general decreasing trend of -2.09 over the 20 years period with only HL showed an increasing trend of + 2.80. There was CR difference between the 11 administrative divisions of Sarawak, and it warrants more detailed population data for confirmation.

## Supplementary Information

**Additional file 1: **Cancer Notification Form. World Standard Population (Segi World Standard Population).** Supplementary Table 1.** Specific haematological cancer categories that were obtained from ICD-10 and ICD-O.** Supplementary Table 2.** The incidence in Sarawak 1996 to 2015 according to the ethnic groups.** Supplementary Table 3.** Incidence of specific haematological cancer categories in Sarawak 1996 to 2015.** Supplementary Table 4.** Summary of six selected specific haematological cancer categories in Sarawak 1996 to 2015.

## Data Availability

The data that support the findings of this study are available from Sarawak State Health Department but restrictions apply to the availability of these data, which were used under license for the current study, and so are not publicly available. Data are however available from the authors upon reasonable request and with permission of Sarawak State Health Department (Dr Ooi Choo Huck).

## Abbreviations

AL: Acute leukaemia
ALL: Acute lymphoblastic leukaemia
AML: Acute myeloid leukaemia and related precursor neoplasms
APML: Acute promyelocytic leukaemia
ASR: Age-standardised Incidence Rate
CML: Chronic myeloid leukaemia
CLL/SLL: Chronic lymphocytic leukaemia/small lymphocytic lymphoma
CNF: Cancer Notification Form
CR: Crude Rate
CR74: Cumulative rate until completion of 74 years of age
CumR: Cumulative Risk
HL: Hodgkin lymphoma
IC: National Identification Card
IR: Incidence Rate
MDS: Myelodysplastic syndrome
NHL: Non-Hodgkin lymphoma
PCN: Plasma cell neoplasm
PR: Permanent residents
SE: Standard Error
SCR: Sarawak Cancer Registry
95% CL: 95% Confidence Limit of ASR

## References

[1] Government S. The Geography of Sarawak [Available from: https://sarawak.gov.my/web/home/article_view/159/176/?id=151.

[2] Ministry of Tourism CIPAS. Sarawak Tourism Quick Facts 2021 2021. Available from: https://mtcp.sarawak.gov.my/upload/file_folder/2022/Sarawak%20Tourism%20Quick%20Facts%202021.pdf.

[3] Department SPUoCMs. Sarawak Facts and Figures 2012: Sarawak Government; 2012. Available from: https://sarawak.gov.my/ebook/Fact_and_Figures_2012/files/basic-html/index.html.

[4] Ooi CH, Yao SK, Usop AKD, Bakar H, Wahab M, Assan J. Epidemiology of Cancer in Sarawak 1996 to 2000. Sarawak: Sarawak Health Department; 2005.

[5] Ooi CH, Wahab M. Epidemiology of Cancer in Sarawak 2001–2005. Sarawak: Sarawak Health Department; 2009.

[6] Segi M. Cancer mortality for selected sites in 24 countries. Nagoya, Japan: Japan Cancer Society; 1950.

[7] Population by state, administrative district and sex, 2016 - 2018. Available from: https://www.dosm.gov.my/v1/index.php?r=column3/accordion&menu_id=amZNeW9vTXRydTFwTXAxSmdDL1J4dz09. Cited 6th Sept 2022.

[8] Chang YM, Swaran Y, Phoon YK, Sothirasan K, Sim HT, Lim KB (2009). Haplotype diversity of 17 Y-chromosomal STRs in three native Sarawak populations (Iban, Bidayuh and Melanau) in East Malaysia. Forensic Sci Int Genet.

[9] Linton RE, Daker M, Khoo AS, Choo DCY, Viljoen M, Neilsen PM (2021). Nasopharyngeal carcinoma among the Bidayuh of Sarawak, Malaysia: History and risk factors. Oncol Lett.

[10] Devi BC, Pisani P, Tang TS, Parkin DM (2004). High incidence of nasopharyngeal carcinoma in native people of Sarawak Borneo Island. Cancer Epidemiol Biomarkers Prev.

[11] Cancer Incidence in Five Continents, Vol. XI (electronic version) Lyon: International Agency for Research on Cancer; 2017 [Available from: https://ci5.iarc.fr/CI5-XI/Default.aspx.

[12] Dong Y, Shi O, Zeng Q, Lu X, Wang W, Li Y (2020). Leukemia incidence trends at the global, regional, and national level between 1990 and 2017. Exp Hematol Oncol.

[13] Park W-J, Park J-H, Cho S, Shin MG (2021). Twenty-year incidence trend of hematologic malignancies in the Republic of Korea: 1999–2018. Blood Res.

[14] Solans M, Fàbrega A, Morea D, Auñon-Sanz C, Granada I, Roncero JM (2019). Population-based incidence of lymphoid neoplasms: Twenty years of epidemiological data in the Girona province Spain. Cancer Epidemiol.

[15] Kuan JW, Melaine MS (2018). The epidemiology of chronic myeloid leukaemia in southern Sarawak Borneo Island. Med J Malaysia.

[16] Cowan AJ, Allen C, Barac A, Basaleem H, Bensenor I, Curado MP (2018). Global burden of multiple myeloma: a systematic analysis for the global burden of disease study 2016. JAMA Oncol.

[17] Miranda-Filho A, Piñeros M, Znaor A, Marcos-Gragera R, Steliarova-Foucher E, Bray F (2019). Global patterns and trends in the incidence of non-Hodgkin lymphoma. Cancer Causes Control.

[18] Alaggio R, Amador C, Anagnostopoulos I, Attygalle AD, Araujo IBO, Berti E (2022). The 5^th^ edition of the world health organization classification of haematolymphoid tumours: lymphoid neoplasms. Leukemia.

[19] Khoury JD, Solary E, Abla O, Akkari Y, Alaggio R, Apperley JF (2022). The 5^th^ edition of the world health organization classification of haematolymphoid tumours: myeloid and histiocytic/dendritic neoplasms. Leukemia..

