# Supplementary material for: The epidemiology of haematological cancers in Sarawak, Malaysia (1996 to 2015)
Source: BMC Cancer. 2023 Jun 19;23:563. doi: 10.1186/s12885-023-10988-y (PMC10278303; doi:10.1186/s12885-023-10988-y)
Supplement: Supplementary file 1 — Additional file 1: Cancer Notification Form. World Standard Population (Segi World Standard Population). Supplementary Table 1. Specific haematological cancer categories that were obtained from ICD-10 and ICD-O. Supplementary Table 2. The incidence in Sarawak 1996 to 2015 according to the ethnic groups. Supplementary Table 3. Incidence of specific haematological cancer categories in Sarawak 1996 to 2015. Supplementary Table 4. Summary of six selected specific haematological cancer categories in Sarawak 1996 to 2015. [file 12885_2023_10988_MOESM1_ESM.docx]

**Supplementary Data**

Contents

[Cancer Notification Form 2](#_Toc133504268)

[World Standard Population (Segi World Standard Population) 4](#_Toc133504269)

[Supplementary Table 1 5](#_Toc133504270)

[Supplementary Table 2 10](#_Toc133504271)

[Supplementary Table 3 11](#_Toc133504272)

[Supplementary Table 4 12](#_Toc133504273)

# Cancer Notification Form


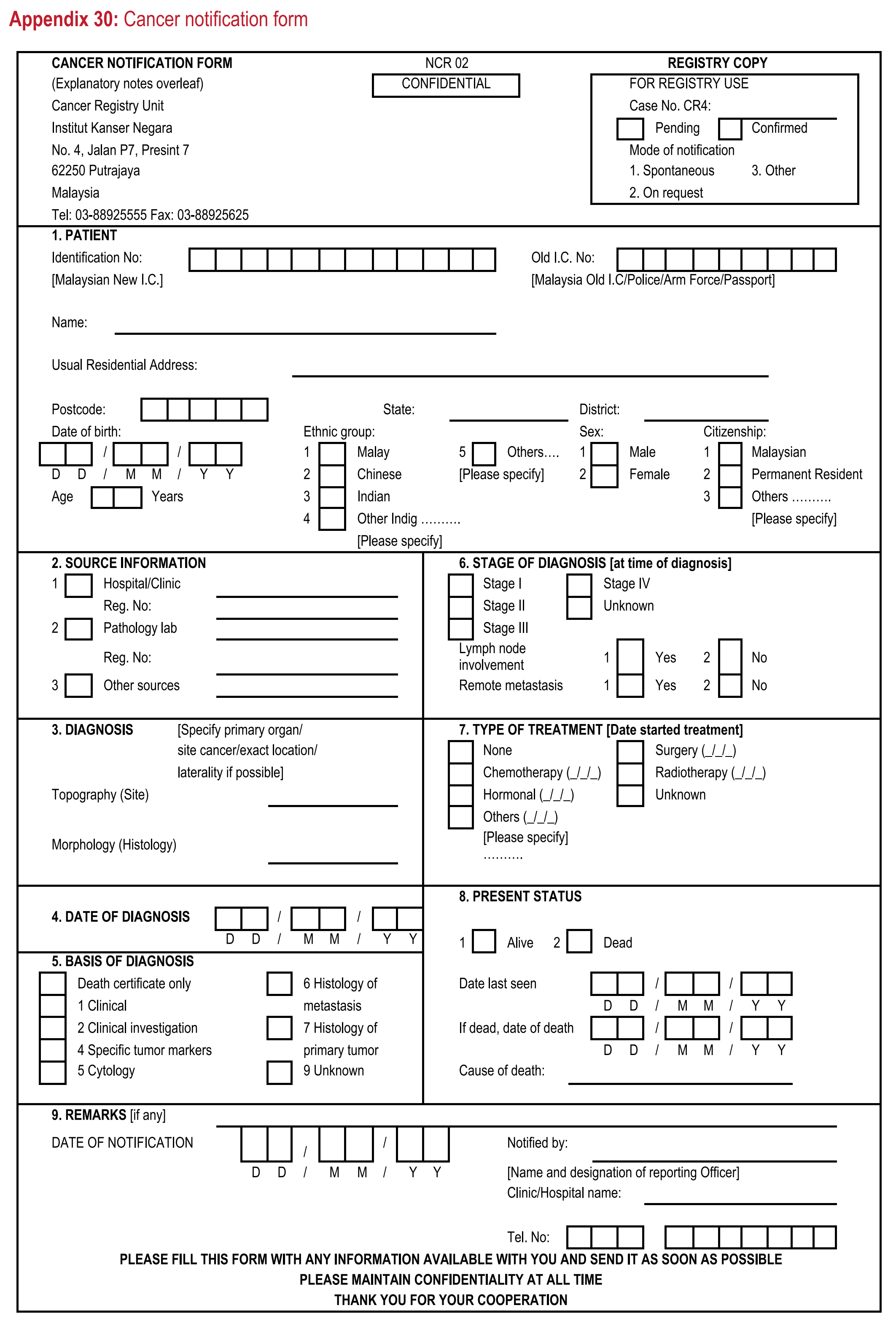


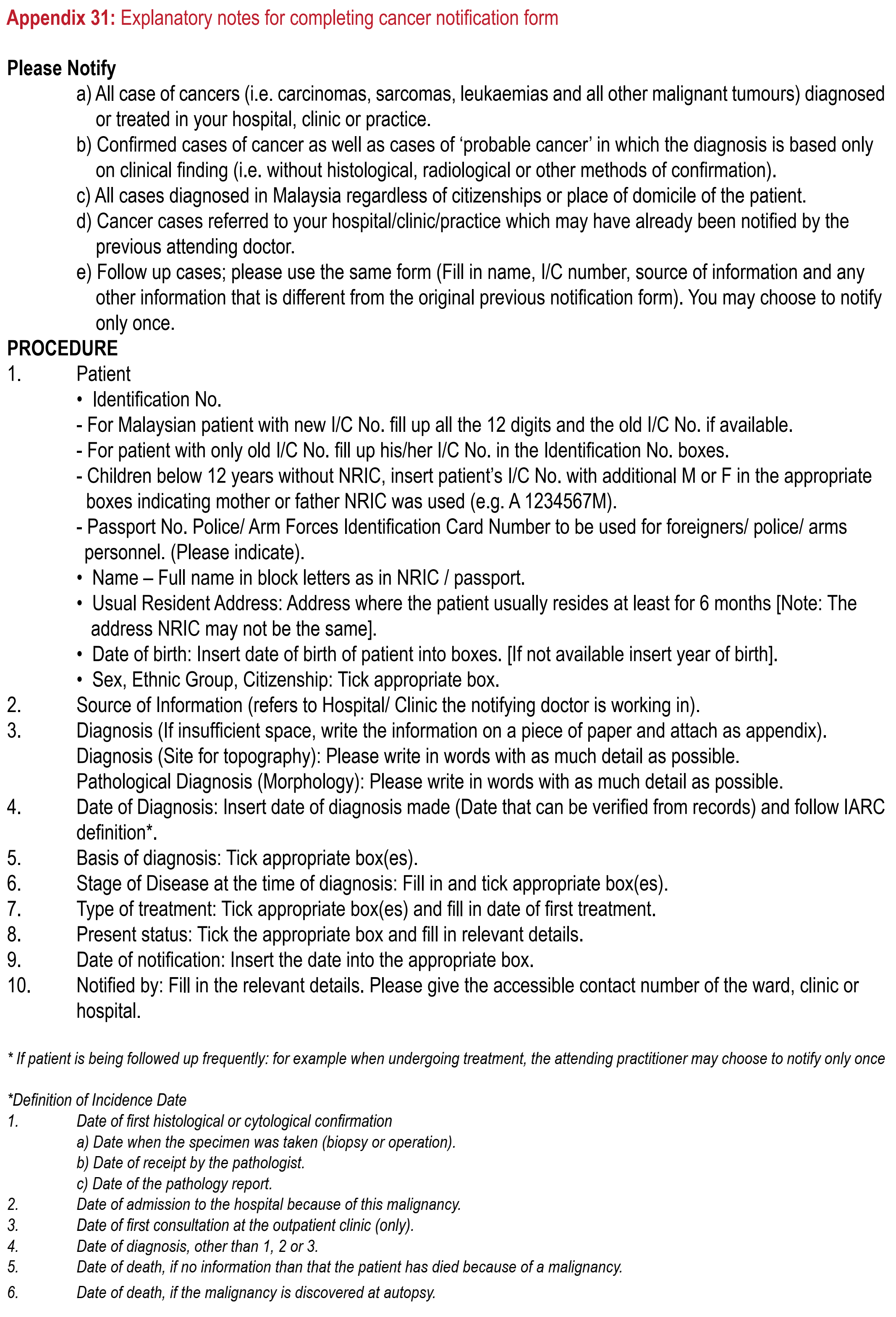


# World Standard Population (Segi World Standard Population)

| Age group index (i) | Age group | Population (w_i_) |
| --- | --- | --- |
| 1 | 0-4 | 12000 |
| 2 | 5-9 | 10000 |
| 3 | 10-14 | 9000 |
| 4 | 15-19 | 9000 |
| 5 | 20-24 | 8000 |
| 6 | 25-29 | 8000 |
| 7 | 30-34 | 6000 |
| 8 | 35-39 | 6000 |
| 9 | 40-44 | 6000 |
| 10 | 45-49 | 6000 |
| 11 | 50-54 | 5000 |
| 12 | 55-59 | 4000 |
| 13 | 60-64 | 4000 |
| 14 | 65-69 | 3000 |
| 15 | 70-74 | 2000 |
| 16 | 75+ | 2000 |
| **Total** | | 100000 |

# Supplementary Table 1

Supplementary Table 1. Specific haematological cancer categories that were obtained from ICD-10 and ICD-O

| No. | Specific haematological cancer categories | ICD-10 | | ICD-O | |
| --- | --- | --- | --- | --- | --- |
|  |  | Code | Name | Code | Name |
|  | Hodgkin lymphoma (HL) | C810  C811  C812  C813  C814  C817  C819 | Nodular lymphocyte predominant Hodgkin lymphoma  Nodular sclerosis (classical) Hodgkin lymphoma  Mixed cellularity (classical) Hodgkin lymphoma  Lymphocyte depleted (classical) Hodgkin lymphoma  Lymphocyte-rich (classical) Hodgkin lymphoma  Other (classical) Hodgkin lymphoma  Hodgkin lymphoma, unspecified | 9659/3  9663/3  9664/3  9665/3  9667/3  9652/3  9653/3  9654/3  9655/3  9651/3  9650/3  9661/3 | Hodgkin lymphoma, nodular lymphocyte predominance  Hodgkin lymphoma, nodular sclerosis, NOS  Hodgkin lymphoma, nodular sclerosis, cellular phase  Hodgkin lymphoma, nodular sclerosis, grade 1  Hodgkin lymphoma, nodular sclerosis, grade 2  Hodgkin lymphoma, mixed cellularity, NOS  "Hodgkin lymphoma, lymphocyte depletion, NOS  Classical Hodgkin lymphoma, lymphocyte depletion, NOS"  Hodgkin lymphoma, lymphocyte depletion, diffuse fibrosis  Hodgkin lymphoma, lymphocyte depletion, reticular  "Hodgkin lymphoma, lymphocyte-rich"  Hodgkin lymphoma, NOS  Hodgkin granuloma [obs] |
|  | HL & non-Hodgkin lymphoma (NHL) |  |  | 9596/3 | Composite Hodgkin and nonHodgkin lymphoma |
|  | NHL/lymphoid leukaemia, not otherwise specified (NOS) | C857  C859  C919 | Other specified types of non-Hodgkin lymphoma  Non-Hodgkin lymphoma, unspecified  Lymphoid leukaemia, unspecified | 9590/3  9591/3  9832/3  9820/3  9970/1 | Malignant lymphoma, NOS  Malignant lymphoma, non-Hodgkin, NOS  Prolymphocytic leukemia, NOS  Lymphoid leukemia, NOS  Lymphoproliferative disorder, NOS |
|  | Mature B cell NHL/leukaemia | C820  C821  C822  C823  C824  C825  C826  C827  C829  C830  C831  C833  C837  C838  C839  C851  C852  C880  C884  C911  C913  C914  C918  C832  C834 | Follicular lymphoma grade I  Follicular lymphoma grade II  Follicular lymphoma grade III, unspecified  Follicular lymphoma grade IIIa  Follicular lymphoma grade IIIb  Diffuse follicle centre lymphoma  Cutaneous follicle centre lymphoma  Other types of follicular lymphoma  Follicular lymphoma, unspecified  Small cell B-cell lymphoma  Mantle cell lymphoma  Diffuse large B-cell lymphoma  Burkitt lymphoma  Other non-follicular lymphoma  Non-follicular (diffuse) lymphoma, unspecified  B-cell lymphoma, unspecified  Mediastinal (thymic) large B-cell lymphoma  Waldenström macroglobulinaemia  Extranodal marginal zone B-cell lymphoma of mucosa-associated lymphoid tissue [MALT-lyphoma]  Chronic lymphocytic leukaemia of B-cell type  Prolymphocytic leukaemia of B-cell type  Hairy-cell leukaemia  Mature B-cell leukaemia Burkitt-type  Mixed small and large cell (diffuse)  Immunoblastic (diffuse) | 9695/3  9691/3  9698/3  9597/3  9675/3  9690/3  9671/3  9689/3  9670/3  9673/3  9680/3  9684/3  9688/3  9735/3  9737/3  9687/3  9678/3  9712/3  9766/1  9767/1  9768/1  9679/3  9738/3  9761/3  9699/3  9823/3  9833/3  9940/3  9826/3 | Follicular lymphoma, grade 1  Follicular lymphoma, grade 2  "Follicular lymphoma, grade 3  "  Primary cutaneous follicle centre lymphoma  Malignant lymphoma, mixed small and large cell, diffuse [obs] (see also M-9690/3)  Follicular lymphoma, NOS (see also M-9675/3)  Malignant lymphoma, lymphoplasmacytic (see also M-9761/3)  Splenic marginal zone B-cell lymphoma (C42.2)  Malignant lymphoma, small B lymphocytic, NOS (see also M-9823/3)  Mantle cell lymphoma (includes all variants: blastic, pleomorphic, small cell)  Malignant lymphoma, large B-cell, diffuse, NOS  Malignant lymphoma, large B-cell, diffuse, immunoblastic, NOS  T-cell/histiocyte rich large B-cell lymphoma  Plasmablastic lymphoma  ALK positive large B-cell lymphoma  Burkitt lymphoma, NOS (see also M-9826/3) (includes all variants)  Primary effusion lymphoma  Intravascular large B-cell lymphoma (C49.9)  Angiocentric immunoproliferative lesion  Angioimmunoblastic lymphadenopathy (AIC)  T-gamma lymphoproliferative disease  Mediastinal large B-cell lymphoma (C38.3)  Large B-cell lymphoma arising in HHV8-associated multicentric Castleman disease  Waldenstrom macroglobulinemia (C42.0) (see also M-9671/3)  Marginal zone B-cell lymphoma, NOS  B-cell chronic lymphocytic leukemia/small lymphocytic lymphoma (see also M-9670/3)  Prolymphocytic leukemia, B-cell type  Hairy cell leukemia (C42.1)  Burkitt cell leukemia (see also M-9687/3) |
|  | Mature T cell NHL/leukaemia | C840  C841  C844  C845  C846  C847  C848  C849  C860  C861  C862  C863  C864  C865  C866  C915  C916  C917 | Mycosis fungoides  Sézary disease  Peripheral T-cell lymphoma, not elsewhere classified  Other mature T/NK-cell lymphomas  Anaplastic large cell lymphoma, ALK-positive  Anaplastic large cell lymphoma, ALK-negative  Cutaneous T-cell lymphoma, unspecified  Mature T/NK-cell lymphoma, unspecified  Extranodal NK/T-cell lymphoma, nasal type  Hepatosplenic T-cell lymphoma  Enteropathy-type (intestinal) T-cell lymphoma  Subcutaneous panniculitis-like T-cell lymphoma  Blastic NK-cell lymphoma  Angioimmunoblastic T-cell lymphoma  Primary cutaneous CD30-positive T-cell proliferations  Adult T-cell lymphoma/leukaemia [HTLV-1-associated]  Prolymphocytic leukaemia of T-cell type  Other lymphoid leukaemia | 9700/3  9701/3  9702/3  9724/3  9725/3  9726/3  9714/3  9709/3  9719/3  9716/3  9717/3  9708/3  9705/3  9718/3  9827/3  9834/3  9831/3  9948/3 | "Mycosis fungoides (C44._)  "Sezary syndrome  Mature T-cell lymphoma, NOS  Systemic EBV positive T-cell lymphoproliferative disease of childhood  Hydroa vacciniforme-like lymphoma  Primary cutaneous gammadelta T-cell lymphoma  Anaplastic large cell lymphoma, T cell and Null cell type  Cutaneous T-cell lymphoma, NOS (C44._)  NK/T-cell lymphoma, nasal and nasal-type  Hepatosplenic T-cell lymphoma  Intestinal T-cell lymphoma  Subcutaneous panniculitislike T-cell lymphoma  Angioimmunoblastic T-cell lymphoma  Primary cutaneous CD30+ T-cell lymphoproliferative disorder (C44._)  Adult T-cell leukemia/lymphoma (HTLV-1 positive) (includes all variants)  Prolymphocytic leukemia, T-cell type  T-cell large granular lymphocytic leukemia  Aggressive NK-cell leukemia |
|  | Plasma cell neoplasm and other diseases with paraprotein (PCN) | C882  C883  C887  C889  C900  C901  C902  C903 | Other heavy chain disease  Immunoproliferative small intestinal disease  Other malignant immunoproliferative diseases  Malignant immunoproliferative disease, unspecified  Multiple myeloma  Plasma cell leukaemia  Extramedullary plasmacytoma  Solitary plasmacytoma | 9762/3  9764/3  9765/1  9769/1  9760/3  9732/3  9733/3  9734/3  9731/3 | Heavy chain disease, NOS  Immunoproliferative small intestinal disease (C17._)  Monoclonal gammopathy of undetermined significance  Immunoglobulin deposition disease  Immunoproliferative disease, NOS  Multiple myeloma (C42.1)  Plasma cell leukemia (C42.1)  Plasmacytoma, extramedullary (not occurring in bone)  Plasmacytoma, NOS |
|  | Acute leukaemia (AL), NOS | C947  C950 | Other specified leukaemias  Acute leukaemia of unspecified cell type | 9801/3 | Acute leukemia, NOS |
|  | Acute myeloid leukaemia & related precursor neoplasms (AML), which includes acute promyelocytic leukaemia (APML) | C920  C923  C924  C925  C926  C927  C928  C930  C940  C942  C944 | Acute myeloblastic leukaemia [AML]  Myeloid sarcoma  Acute promyelocytic leukaemia [PML]  Acute myelomonocytic leukaemia  Acute myeloid leukaemia with 11q23-abnormality  Other myeloid leukaemia  Acute myeloid leukaemia with multilineage dysplasia  Acute monoblastic/monocytic leukaemia  Acute erythroid leukaemia  Acute megakaryoblastic leukaemia  Acute panmyelosis with myelofibrosis | 9861/3  9872/3  9873/3  9874/3  9896/3  9930/3  9866/3  9867/3  9871/3  9897/3  9865/3  9869/3  9895/3  9891/3  9840/3  9910/3  9911/3  9931/3  9870/3 | Acute myeloid leukemia, NOS (see also M-9930/3) (FAB or WHO type not specified)  Acute myeloid leukemia, minimal differentiation  Acute myeloid leukemia without maturation  Acute myeloid leukemia with maturation  Acute myeloid leukemia, t(8;21)(q22;q22)  Myeloid sarcoma (see also M-9861/3)  Acute promyelocytic leukemia, t(15;17)(q22;q11-12)  Acute myelomonocytic leukemia  Acute myeloid leukemia with abnormal marrow eosinophils (includes all variants)  Acute myeloid leukemia, 11q23 abnormalities  Acute myeloid leukemia with t(6;9) (p23;q34); DEK-NUP214  Acute myeloid leukemia with inv(3)(q21;q26.2) or t(13.3)(q21;q26.2); RPN1-EVI1  Acute myeloid leukemia with myelodysplasia-related changes  Acute monocytic leukemia  Acute myeloid leukemia, M6 type  Acute megakaryoblastic leukemia  Acute myeloid leukemia (megakaryoblastic) with t(1;22)(p13;q13); RBM15-MKL1  Acute panmyelosis with myelofibrosis (C42.1)  Acute basophilic leukemia |
|  | Precursor lymphoid neoplasms, i.e. acute lymphoblastic leukaemia (ALL) | C835  C910 | Lymphoblastic (diffuse) lymphoma  Acute lymphoblastic leukaemia [ALL] | 9727/3  9728/3  9729/3  9811/3  9812/3  9813/3  9814/3  9815/3  9816/3  9817/3  9818/3  9835/3  9836/3  9837/3 | Precursor cell lymphoblastic lymphoma, NOS (see also M-9835/3)  Precursor B-cell lymphoblastic lymphoma (see also M-9836/3)  Precursor T-cell lymphoblastic lymphoma (see also M-9837/3)  B lymphoblastic leukemia/lymphoma, NOS  B lymphoblastic leukemia/lymphoma with t(9;22)(q34;q11.2); BCR-ABL1  B lymphoblastic leukemia/lymphoma with t(v;11q23); MLL rearranged  B lymphoblastic leukemia/lymphoma with t(12;21)(p13;q22);TEL-AML1 (ETV6-RUNX1)  B lymphoblastic leukemia/lymphoma with hyperdiploidy  B lymphoblastic leukemia/lymphoma with hypodiploidy (Hypodiploid ALL)  B lymphoblastic leukemia/lymphoma with t(5;14)(q31;q32); IL3-IGH  B lymphoblastic leukemia/lymphoma with t(1;19)(q23;p13.3);E2A-PBX1 (TCF3-PBX1)  Precursor cell lymphoblastic leukemia, NOS (see also M-9727/3)  Precursor B-cell lymphoblastic leukemia (see also M-9728/3)  Precursor T-cell lymphoblastic leukemia (see also M-9729/3) |
|  | AL of ambiguous lineage |  |  | 9805/3  9806/3  9807/3  9808/3  9809/3 | Acute biphenotypic leukemia  Mixed phenotype acute leukemia with t(9;22)(q34;q11.2); BCR-ABL1  Mixed phenotype acute leukemia with t(v;11q23); MLL rearranged  Mixed phenotype acute leukemia, B/myeloid, NOS  Mixed phenotype acute leukemia, T/myeloid, NOS |
|  | Myeloproliferative neoplasm (MPN), which includes chronic myeloid leukaemia (CML) and juvenile myelomonocytic leukaemia (JMML) | C921  C933 | Chronic myeloid leukaemia [CML], BCR/ABL-positive  Juvenile myelomonocytic leukaemia | 9863/3  9875/3  9950/3  9960/3  9961/3  9962/3  9963/3  9964/3  9946/3  9975/3 | Chronic myeloid leukemia, NOS  Chronic myelogenous leukemia, BCR/ABL positive  Polycythemia vera  Myeloproliferative neoplasm, NOS  Primary myelofibrosis  Essential thrombocythemia  Chronic neutrophilic leukemia  Chronic eosinophilic leukemia, NOS  Juvenile myelomonocytic leukemia  Myloproliferative neoplasm, unclassifiable |
|  | Myelodysplastic syndrome (MDS) |  |  | 9980/3  9982/3  9983/3  9984/3  9985/3  9986/3  9989/3  9991/3  9992/3 | Refractory anemia  Refractory anemia with sideroblasts  Refractory anemia with excess blasts  Refractory anemia with excess blasts in transformation [obs]  Refractory cytopenia with multilineage dysplasia  Myelodysplastic syndrome with 5q deletion (5q-) syndrome  Myelodysplastic syndrome, NOS  Refractory neutropenia  Refractory thrombocytopenia |
|  | Myelodysplastic/myeloproliferative neoplasms (MDS/MPN), which includes chronic myelomonocytic leukaemia (CMML) | C922  C931  C946 | Atypical chronic myeloid leukaemia, BCR/ABL- negative  Chronic myelomonocytic leukaemia  Myelodysplastic and myeloproliferative disease, not elsewhere classified | 9876/3  9945/3 | "Atypical chronic myeloid leukemia, BCR/ABL negative  Chronic myelomonocytic leukemia, NOS |
|  | Myeloid/lymphoid neoplasm with eosinophilia and gene rearrangement |  |  | 9965/3  9966/3  9967/3 | Myeloid and lymphoid neoplasms with PDGFRA rearrangement  Myeloid neoplasms with PDGFRB rearrangement  Myeloid and lymphoid neoplasms with FGFR1 abnormalities |
|  | Secondary myeloid neoplasm, which includes therapy-related myeloid neoplasm |  |  | 9898/1  9898/3  9920/3  9987/3 | Transient abnormal myelopoiesis  Myeloid leukemia associated with Down Syndrome  Therapy related myeloid neoplasm  Therapy-related myelodysplastic syndrome, NOS |
|  | Histiocyte and dendritic cell neoplasm | C960  C964  C965  C966  C968  C961  C963 | Multifocal and multisystemic (disseminated) Langerhans-cell histiocytosis [Letterer-Siwe disease]  Sarcoma of dendritic cells (accessory cells)  Multifocal and unisystemic Langerhans-cell histiocytosis  Unifocal Langerhans-cell histiocytosis  Histiocytic sarcoma  Malignant histiocytosis  True histiocytic lymphoma | 9751/3  9750/3  9757/3  9756/3  9758/3  9759/3  9755/3  9754/3 | Langerhans cell histiocytosis, NOS (This code for all types of Langerhans cell histiocytosis replaces the former 9751/1 through 9754/3 codes)  Malignant histiocytosis  Interdigitating dendritic cell sarcoma  Langerhans cell sarcoma  Follicular dendritic cell sarcoma  Fibroblastic reticular cell tumor  Histiocytic sarcoma  Langerhans cell histiocytosis, disseminated (use 9751/3) [obs] |
|  | Myeloid leukaemia, NOS | C929  C937  C939 | Myeloid leukaemia, unspecified  Other monocytic leukaemia  Monocytic leukaemia, unspecified | 9860/3 | Myeloid leukemia, NOS |
|  | Mastocytosis | C943  C962 | Mast cell leukaemia  Malignant mast cell tumour | 9742/3  9740/3  9740/1  9741/1 | Mast cell leukemia (C42.1)  Mast cell sarcoma  Mastocytoma, NOS  Indolent systemic mastocytosis |
|  | Leukaemia, NOS | C951  C957  C959 | Chronic leukaemia of unspecified cell type  Other leukaemia of unspecified cell type  Leukaemia, unspecified | 9800/3 | Leukemia, NOS |
|  | Lymphoid proliferations and lymphomas associated with immune deficiency and dysregulation (L-ID) |  |  | 9971/1  9971/3 | Post transplant lymphoproliferative disorder, NOS  Polymorphic post transplant lymphoproliferative disorder |
|  | Haematological neoplasm, NOS | C967  C969 | Other specified malignant neoplasms of lymphoid, haematopoietic and related tissue  Malignant neoplasm of lymphoid, haematopoietic and related tissue, unspecified |  |  |

# Supplementary Table 2

Supplementary Table 2. The incidence in Sarawak 1996 to 2015 according to the ethnic groups

| Ethnic group | Incidence | % |
| --- | --- | --- |
| Malay | 929 | 23.5 |
| Chinese | 1119 | 28.4 |
| Indian | 8 | 0.2 |
| Thai | 0 | 0.0 |
| Kadazan | 0 | 0.0 |
| Melanau | 132 | 3.3 |
| Murut | 8 | 0.2 |
| Bajau | 0 | 0.0 |
| Bidayuh | 402 | 10.2 |
| Iban | 1118 | 28.3 |
| Kayan | 46 | 1.2 |
| Kenyah | 35 | 0.9 |
| Kelabit | 6 | 0.2 |
| Bisayah Sarawak | 11 | 0.3 |
| Kedayan Sarawak | 10 | 0.3 |
| Lun Bawang | 18 | 0.5 |
| Berawan | 2 | 0.1 |
| Penan | 6 | 0.2 |
| Punan | 2 | 0.1 |
| Jawa Sarawak | 1 | 0.0 |
| Other Indigenous Sarawak | 61 | 1.5 |
| Others | 29 | 0.7 |
| Unknown/missing data | 4 | 0.1 |
| TOTAL | **3947** | **100.0** |

“Others” stands for other ethnic groups not encoded/listed above.

# Supplementary Table 3

Supplementary Table 3. Incidence of specific haematological cancer categories in Sarawak 1996 to 2015

| Disease category | | | All | Paed | Adult |
| --- | --- | --- | --- | --- | --- |
|  | Lymphoma | Hodgkin lymphoma (HL) | 275 | 29 | 246 |
|  |  | HL & non-Hodgkin lymphoma (NHL) | 0 | 0 | 0 |
|  |  | NHL/lymphoid leukaemia, not otherwise specified (NOS) | 1277 | 77 | 1200 |
|  |  | Mature B cell NHL/leukaemia | 553 | 32 | 521 |
|  |  | Mature T cell NHL/leukaemia | 75 | 8 | 67 |
|  | PCN | Plasma cell neoplasm and other diseases with paraprotein (PCN) | 279 | 0 | 279 |
|  | AL | Acute leukaemia (AL), NOS | 67 | 21 | 46 |
|  |  | Acute myeloid leukaemia & related precursor neoplasms (AML), which includes acute promyelocytic leukaemia (APML) | 596 | 153 | 443 |
|  |  | Precursor lymphoid neoplasms, i.e. acute lymphoblastic leukaemia (ALL) | 551 | 400 | 151 |
|  |  | AL of ambiguous lineage | 0 | 0 | 0 |
|  | MPN | Myeloproliferative neoplasm (MPN), which includes chronic myeloid leukaemia (CML) and juvenile myelomonocytic leukaemia (JMML) | 206 | 6 | 200 |
|  | MDS | Myelodysplastic syndrome (MDS) | 0 | 0 | 0 |
|  | MDS/MPN | Myelodysplastic/myeloproliferative neoplasms (MDS/MPN), which includes chronic myelomonocytic leukaemia (CMML) | 2 | 0 | 2 |
|  |  | Myeloid/lymphoid neoplasm with eosinophilia and gene rearrangement | 0 | 0 | 0 |
|  |  | Secondary myeloid neoplasm, which includes therapy-related myeloid neoplasm | 0 | 0 | 0 |
|  |  | Histiocyte and dendritic cell neoplasm | 9 | 6 | 3 |
|  |  | Myeloid leukaemia, NOS | 11 | 2 | 9 |
|  |  | Mastocytosis | 0 | 0 | 0 |
|  |  | Leukaemia, NOS | 46 | 15 | 31 |
|  |  | Lymphoid proliferations and lymphomas associated with immune deficiency and dysregulation (L-ID) | 0 | 0 | 0 |
|  |  | Haematological neoplasm, NOS | 0 | 0 | 0 |
| TOTAL | | | **3947** | **749** | **3198** |

# Supplementary Table 4

Supplementary Table 4. Summary of six selected specific haematological cancer categories in Sarawak 1996 to 2015

| Category | Summary | Total | Male | Female | M:F ratio |
| --- | --- | --- | --- | --- | --- |
| AML | Incidence | 596 | 307 | 289 | 1.1 |
|  | CR* | 1.3 | 1.4 | 1.3 | **1.0** |
|  | CR74 | 0.1 | 0.1 | 0.1 | 1.0 |
|  | ASR* | 1.5 | 1.5 | 1.4 | 1.0 |
| ALL | Incidence | 551 | 316 | 235 | 1.3 |
|  | CR* | 1.2 | 1.4 | 1.1 | **1.3** |
|  | CR74 | 0.1 | 0.1 | 0.1 | 1.4 |
|  | ASR* | 1.3 | 1.4 | 1.1 | 1.3 |
| Mature B cell NHL/leukaemia | Incidence | 553 | 323 | 230 | 1.4 |
|  | CR* | 1.3 | 1.4 | 1.1 | **1.4** |
|  | CR74 | 0.2 | 0.2 | 0.1 | 1.3 |
|  | ASR* | 1.5 | 1.7 | 1.3 | 1.4 |
| HL | Incidence | 275 | 165 | 110 | 1.5 |
|  | CR* | 0.6 | 0.7 | 0.5 | **1.5** |
|  | CR74 | 0.1 | 0.1 | 0.0 | 1.6 |
|  | ASR* | 0.6 | 0.8 | 0.5 | 1.5 |
| PCN | Incidence | 279 | 169 | 110 | 1.5 |
|  | CR* | 0.6 | 0.8 | 0.5 | **1.5** |
|  | CR74 | 0.1 | 0.1 | 0.1 | 1.7 |
|  | ASR* | 0.8 | 0.9 | 0.6 | 1.5 |
| CML | Incidence | 204 | 127 | 77 | 1.6 |
|  | CR* | 0.5 | 0.6 | 0.4 | **1.6** |
|  | CR74 | 0.1 | 0.1 | 0.0 | 1.5 |
|  | ASR* | 0.5 | 0.6 | 0.4 | 1.6 |

*per 100,000 population
